# Cancers preventive practice and the determinants in Amhara regional state, Northwest Ethiopia

**DOI:** 10.1371/journal.pone.0267189

**Published:** 2022-05-19

**Authors:** Getasew Mulat Bantie, Koku Sisay Tamirat, Ashenafi Abate Woya, Amare Alemu Melese, Agumas Fentahun Ayalew, Gizachew Asmare Wubetu, Gizachew Tadesse Wassie, Kalkidan Worku Mitiku, Minichil Genet Minale, Amanuel Addisu Dessie, Selam Fisiha Kassa, Girum Meseret Ayenew

**Affiliations:** 1 Community Health Faculty, Alkan Health Science, Business and Technology College, Bahir Dar City, Ethiopia; 2 Department of Epidemiology and Biostatistics, University of Gondar, School of Public Health, Gondar Town, Ethiopia; 3 Statistics Department, Bahir Dar University, Science College, Bahir Dar City, Ethiopia; 4 Food Safety, and Microbiology Reference Laboratory, Ethiopian Public Health Institute, Addis Ababa, Ethiopia; 5 Department of Epidemiology, Injibara University, College of Health Sciences, Injibara, Ethiopia; 6 Community Health Faculty, Alkan Health Science, Business and Technology College, Bahir Dar city, Ethiopia; 7 Department of Biostatistics and Epidemiology, Bahir Dar University, Bahir Dar, Ethiopia; 8 Department of Reproductive Health, Debre Markos University, Debre Markos, Ethiopia; 9 Bahir Dar Health Science College, Bahir Dar, Ethiopia; 10 Woldia University, School of Public Health, Woldia, Ethiopia; 11 Department of Pediatrics and Child Health Nursing, University of Gondar, College of Medicine and Health Science, School of Nursing, Gondar, Ethiopia; 12 Department of Nutrition and Dietetics, Amhara Public Health Institute, School of Public Health, College of Medicine and Health Science, Bahir Dar University, Bahir Dar, Ethiopia; ICMR-National Institute for Research in Tuberculosis: National Institute of Research in Tuberculosis, INDIA

## Abstract

**Background:**

Cancer is the leading cause of morbidity and mortality globally. In Ethiopia, 5.8% of deaths are attributed to cancer. Therefore, this study aimed to examine the cancers preventive practice and associated factors in North West Ethiopia, 2019.

**Methods:**

A community-based cross-sectional study was conducted among Bahir Dar city residents. A multistage sampling technique was used to select 845 study participants. Data were collected through a validated interviewer administered questionnaire. The questionnaire was adapted from the American cancer association cancer prevention toolkit. Descriptive statistics were computed and presented in charts and texts. The model fitness was checked using Hosmer and Lemeshow goodness of fit (P > 0.05). Bivariable and multivariable logistic regressions were used to identify factors associated with cancer preventive practice. A p-value < 0.2 at bivariate analysis was candidate variables for multivariable logistic regression analysis. Finally, p-value of < 0.05 was considered as a statistically significant predictor for cancer preventive practice at the 95% confidence interval.

**Result:**

A total of 845 study participants took part in the study. Nearly 63% of the respondents were females. About 28% (95%CI: 24, 30) of the study participants had good preventive practice. Age ≥ 45 years (AOR = 0.31; 95%CI: 0.15, 0.62), female (AOR = 0.50, 95%CI: 0.35, 0.71) family member with cancer (AOR = 1.68, 95%CI: 1.07, 2.62) and had good knowledge (AOR = 1.66, 95%CI: 1.14, 2.42) were the identified determinants of cancer preventive practices.

**Conclusion:**

This study revealed that the level of cancer preventive practices was low. Family member with cancer, knowledge about cancer, older age, and being female were significantly associated with cancer preventive practices. This finding underscores the importance of interventions to enhance cancer preventive practices.

## Background

The World Health Organization (WHO) reported that nearly 18.1 million new cases and 9.6 million deaths had occurred worldwide due to cancer [1]. In low-and middle-income countries, approximately 70% of deaths are attributed to cancer [1,2]. In Africa, cancer is an emerging public health issue with estimates of 715, 000 new cases and 542,000 deaths in 2008 [1–3].

A third of cancer deaths in Africa are potentially preventable [4]. Many are because of chronic infections, the aging and growth of the population, and the changing prevalence of risk factors associated with a social and economic transition (including smoking, alcohol consumption, obesity, physical inactivity, and unhealthy diets) [1–3,5–12].

Moreover, according to the WHO Ethiopian cancer mortality profile 2014, 691,000 cancer-related deaths occurred [1]. From these, in females, a total of 59.1% of deaths was related to cancer. Breast cancer 24.4%, Cervical 17.5%, Ovary 7.2%, Leukemia 5.3%, and Colorectal 4.8%, which means only 40.8% of deaths, are attributed to any other causes [13].

The fast-growing, aging population and the surge in the prevalence of the risk factors, such as tobacco use, obesity, alcohol use, unhealthy diet, physical inactivity, and the changes in reproductive health patterns related to urbanization, have been increasing the cancer burden in Ethiopia since recent years. Recognizing this threat, the Ethiopian government has launched a national cancer control plan in 2015. The plan set ambitious objectives to expand a range of preventive interventions, screening tests for early detection, and diagnosis and treatment. It aims to increase cancer awareness of general population by 50%, to reduce the prevalence of tobacco smoking by 30%, overweight and obesity by 5%, prevalence of insufficient physical activity by 10%, prevalence of harmful use of alcohol by 5%, to increase in mean population intake of fruits and vegetables at least twice per week by 15%, and to achieve 80% coverage of vaccine against HPV for girls aged 9 to 13, in 2020 [12]. The Ethiopian cancer control policy urgently requires a clarification of the discrepancies which now exist between ideal levels of public awareness and preventive practice about cancer risk factors and current reality on preventive practices as well [14]. To contribute to the urgent need for the policy, this study aimed to determine the cancer preventive practice level and the determinants among Bahir Dar city residents, northwest Ethiopia.

## Methods

### Study Design, period and setting

A community-based cross-sectional study was employed among adults (18 years and above) of Bahir Dar city residents from May to June 2019. The study setting Bahir Dar city is the capital of Amhara National Regional State which has nine sub-cities. The total population of the city is estimated to be 274,459. Of these, 136,730 were females [15].

### Population and sample

The sample size was determined using a single population proportion formula with the assumptions of cancer preventive practice (50%), 5% margin of error, 10% non-response rate, and a design effect of 2 yielded a final sample size of 845.

A multistage sampling technique was used to recruit the study participants from nine sub-cities.

Initially, Hidar 11, Fasilo, Tana, and Belay Zeleke sub-cities were randomly selected by using a lottery method.

Then, proportional to size allocation was applied, and a systematic sampling technique was used. Accordingly, 201 (Hidar 11), 190 (Fasilo), 129 (Tana), and 325 (Belay Zeleke) participants were selected from the four sub-cities, respectively. Whenever more than one eligible family member was in a household, only one individual was chosen for study using a lottery method.

### Data collection procedure

A pretested, interviewer-administered structured Amharic questionnaire was used to determine cancer-preventive practice. The questionnaire was adapted from the American cancer association tool kit and was slightly modified and translated to the Amharic language. The questionnaire had three components; socio-demographic, knowledge about cancer, and cancer preventive-practice- related characteristics. All enumerators secured written consent from participants before collecting the data. To maintain the quality of the data and to assure that all team members were able to administer the questionnaires uniformly, one day of rigorous training was provided to four nurses with bachelor’s degrees (data collectors) and to two supervisors with master’s degrees.

Enumerators and the supervisors carried out role-plays and had field pre-test activities. On the afternoon of every data collection day, the supervisor assessed each questionnaire and gave feedback to the enumerators. The internal consistency (Cronbach alpha) level of the pretest of cancer preventive practice was 0.86.

### Operational definitions

#### Cancer preventive practice

Those respondents who scored mean and above of the cancer-preventive practice assessing questions correctly were considered as having good practice. Otherwise, they were considered as having poor practices.

#### Knowledge about cancer

Those respondents who scored mean and above of the knowledge assessing questions correctly were considered as having good knowledge about cancer. Otherwise, they were considered as having poor knowledge.

#### A unit of alcohol

Is one small measure of spirits, half a pint of lager (3–4% strength), or half a small glass (175ml) of wine (12% strength).

### Data processing and analysis

Data entry, cleaning, and coding were performed using Epi Data version 4.12 and analyzed using SPSS version 23.0 software. Descriptive statistics were computed. A logistic regression model was used to identify the association between explanatory and outcome variables. Multi-collinearity was checked for the data. The model fitness was checked using Hosmer and Lemeshow goodness of fit (P> 0.05). Predictor variables having p-values < 0.20 were taken into a multivariable logistic regression analysis to see associations between dependent and independent variables. P-values less than 0.05 were taken as the identified predictors of cancer preventive practices.

### Ethical Consideration and consent to participate

Ethical approval was obtained from GAMBY Medical and Business College, Research, and health institute. The support letter was obtained from the Bahir Dar city administration. The objective of the study was clarified to the study participants, and they were also notified that they have the right to opt-out of the study at any point in the interview. Then, the written consent was secured from the respondents. The written consent and the data collection tools were documented and kept confidential in a secure place.

## Results

### Socio-demographic characteristics

A total of 845 study participants took part with a response rate of 98%. The median (±IQR) age of the respondents was 27 (22.35) years. Sixty-three percent of the respondents were males. And about forty-six percent of the respondents were married. Nearly one-third of the respondents had attended secondary education. Furthermore, 24.6% of the participants were privately employed, and nearly one-third (32%) of them were living in privately owned houses, respectively (**Table 1**).

**Table 1:** Sociodemographic characteristics of the respondents, Bahir Dar city residents, North West Ethiopia, 2019 (n = 845).

Characteristics	Category	Frequency	Percent
Age	18–29 years	499	59.1
	30–44 years	251	29.7
	≥ 45 years	95	11.2
Sex	Male	313	37.0
	Female	532	63.0
Religion	Christians	677	80.1
	Muslim	168	19.9
Ethnicity	Amhara	780	92.3
	Not Amhara	65	7.7
Marital status	Married	453	53.6
	Unmarried	390	46.2
Level of education	No formal education	166	19.7
	Primary school	129	15.3
	Secondary school	276	32.7
	College and above	272	32.2
Housing condition	Private house	270	32.0
	Government house	88	10.4
	Rental house	249	29.5
	Living with family/friend	236	27.9
Occupation	Private Employee	208	24.6
	Government Employee	146	17.3
	Merchant	106	12.5
	Student	201	23.8
	House Wife	87	10.3
	Unemployed	80	9.5
	Retired	15	1.8
Family member with cancer	Yes	114	13.5
	No	727	86.0
Preferred health facility	Private health facility	285	33.7
	Government Health facility	354	41.9
	Holy Water	167	19.8

### Knowledge about early warning signs of cancer

Unexplained lump or swelling (55.4%), unhealing sore (55.1%), unexplained bleeding (37.8%), and persistent unexplained pain (24.6%) were the most commonly mentioned cancer warning signs by the study participants (**Table 2**).

**Table 2:** Knowledge about the early warning signs of cancer, Bahir Dar city residents, North West Ethiopia, 2019 (n = 845).

Characteristics	Yes N (%)	No N (%)	Don’t Know N (%)
Do you think unexplained bleeding could be a sign of cancer	412 (48.8)	224 (26.5)	201 (23.8)
Do you think a persistent cough or hoarseness could be a sign of cancer	150 (17.8)	491 (58.1)	196 (23.2)
Do you think a persistent change in bowel or bladder habits could be a sign of cancer	109 (12.9)	493 (58.3)	235 (27.8)
Do you think persistent difficulty swallowing could be a sign of cancer	137 (16.2)	467 (55.3)	233 (27.6)
Do you think a change in the appearance of a mole could be a sign of Cancer	114 (13.5)	539 (63.8)	184 (21.8)
Do you think a sore that does not heal could be a sign of cancer	432 (51.1)	248 (29.3)	157 (18.6)
Do you think an unexplained lump or swelling could be a sign of cancer	468 (55.4)	214 (25.3)	155 (18.3)
Do you think persistent unexplained pain could be a sign of cancer	319 (37.8)	280 (33.1)	238 (28.2)
Do you think unexplained weight loss could be a sign of cancer	208 (24.6)	401 (47.5)	226 (26.7)

### Knowledge about cancer risk factors

Regarding knowledge about risk factors, exposure to cigarette smoking (74.9%), infection with human papillomavirus (HPV) (53.1%), and chronic infection with Hepatitis B or C (49.2%) were the most enlisted risk factors. Only one hundred seventy-one (20.2%) of participants had good knowledge about cancer risk factors (**Table 3**).

**Table 3:** Level of knowledge of the study participants toward cancer risk factors among Bahir Dar city residents, North West Ethiopia 2019, (n = 845).

Knowledge characteristics	Strongly disagree	Disagree	Not sure	Agree	Strongly agree
	N (%)	N (%)	N (%)	N (%)	N (%)
Smoking any cigarettes at all is a risk factor for cancer	81 (9.6)	72 (8.5)	51(6)	308 (36.4)	325 (38.5)
Secondhand smoke is a risk factor for cancer	64 (7.6)	169 (20.0)	125 (14.8)	308 (36.4)	171 (20.2)
Drinking more than 1 unit of alcohol a day is a risk factor for cancer	85 (10.1)	247 (29.2)	180 (21.3)	227 (26.9)	96 (11.4)
Eating < 5 portions of fruit and vegetables a day is a risk factor for cancer	137 (16.2)	321 (38.0)	195 (23.1)	141 (16.7)	41 (4.9)
Eating processed meat once a day or more is a risk factor for cancer	61 (7.2)	275 (32.5)	254 (30.1)	192 (22.7)	53 (6.3)
Being overweight (BMI >25) is a risk factor for cancer	42 (5.0)	270 (32.0)	328 (38.8)	128 (15.1)	67 (7.9)
Getting sunburn more than once as a child is a risk factor for cancer	62 (7.3)	294 (34.8)	327 (38.7)	101 (12.0)	51 (6)
Being older age is a risk factor for cancer	110 (13)	314 (37.2)	145 (17.2)	169 (20.0)	91 (10.8)
Having a close relative with cancer is a risk factor for cancer	154 (18.2)	312 (36.9)	199 (23.6)	130 (15.4)	36 (4.3)
Infection with HPV (Human Papilloma Virus) is a risk factor for cancer	51 (6)	126 (14.9)	203 (24.0)	332 (39.3)	117 (13.8)
Physical inactivity is a risk factor for cancer	148 (17.5)	260 (30.8)	223 (26.4)	157 (18.6)	41 (4.9)
Chronic hepatitis B or C infection is a risk factor for cancer	54 (6.4)	99 (11.7)	262 (31.0)	298 (35.3)	118 (14)

### The level of cancer preventive practices

From cancer prevention practices, about 46.5% of participants did slight physical exercises, 51.7% usually sleep 5–8 hours per day, 88% did not smoke cigarettes, and 45.3% never drink alcohol. About 79% of males and females had a routine screening in the past two years. Nearly 49% of males and 44% of females underwent a physical examination for symptoms in the last two years (**Table 4**).

**Table 4:** Cancer preventive practices of the respondents, Bahir Dar city residents, North West Ethiopia 2019, (n = 845).

Variables		Category			Frequency		Percent	
Physical Activity		None			166		19.6	
		Slight			342		40.5	
		Moderate			257		30.4	
		Heavy			64		7.6	
Hours spent in sleep		< 6 Hours			112		13.3	
		6–8 Hours			437		51.7	
		> 8 Hours			287		34.0	
Ever smoked cigarette		Yes			92		10.9	
		No			744		88.0	
Smoke Cigarette		Yes			31		3.7	
		No			65		7.7	
Alcohol intake		Never			383		45.3	
		1–4 Days/Month			347		41.1	
		3–5 Days/Week			66		7.8	
		6–7 Days/Week			38		4.5	
Avoid eating the fat meal		Yes			504		59.6	
		No			330		39.1	
Add more salt to food		Yes			625		74.0	
		No			209		24.7	
Occupational exposure to carcinogens		Yes			276		32.7	
		No			558		66.0	
Occupational exposure to carcinogens		Chemicals			49		5.8	
		Textile Dust			131		15.5	
		Wood dust			31		3.7	
		Gases			24		2.8	
		Solvent			11		1.3	
		Oil			32		3.8	
		X-rays/radiation materials			72		8.5	
Self-breast exam (n = 532)		Yes			135		25.3	
		No			397		74.7	
**Physical examination and screening for cancer in the last 2 years**								
**Sex**	**Types of Examination done**	**Yes**					**No**	
		**For Routine Exams**		**For Symptoms**				
		**Frequency**	**Percent**	**Frequency**		**Percent**	**Frequency**	**Percent**
Male	Colonoscopy	11	3.5	37		11.8	265	84.7
	Sigmoidoscopy	10	3.2	44		14.1	259	82.7
	A Physical Exam	61	19.5	72		23	180	57.5
	Blood Analysis	71	22.7	93		29.7	149	47.6
Female	Colonoscopy	17	3.2	43		5.1	472	88.7
	Sigmoidoscopy	12	2.2	17		2.0	503	94.5
	A Physical Exam	49	9.2	126		14.9	357	67.1
	Blood Analysis	75	14.1	154		18.2	303	56.9
	Pap Smear	37	4.4	42		5.0	438	51.8
	Mammogram	42	5.0	37		4.4	438	51.8

### Factors associated with cancer preventive practices

In the univariate logistic regression analysis, age, sex, occupation, marital status, education, knowledge about cancer risks, and family member with cancer were the factors associated with cancer preventive practice at a 20% level of significance. In the multivariable logistic regression analysis, age, sex, knowledge about cancer risks, and family member with cancer were predictors of cancer preventive practice at a p-value of less than < 0.05.

Thus, for those study participants whose age was above 45 years, the odds of cancer preventive practice were 69% (AOR = 0.31; 95%CI: 0.15, 0.62) lower compared to younger respondents. Similarly, females had a 50% (AOR = 0.50; 95%CI: 0.35, 0.71) of lower chance of cancer preventive practice compared to males. Conversely, for those study participants who had a family member with cancer, the odds of cancer preventive practice was 1.66 (AOR = 1.68; 95%CI: 1.07, 2.62) times higher compared to those who had not. Likewise, for those participants who had good knowledge about cancer risks, the odds of cancer preventive practice were 1.66 (AOR = 1.66; 95%CI: 1.14, 2.42) times higher compared to those who had poor knowledge about cancer risks (**Table 5**).

**Table 5:** Simple logistic regression analysis on factors associated with cancer preventive practice of the respondents, Bahir Dar city residents, Northwest Ethiopia, 2019 (n = 845).

Variables	Cancer-preventive practice		COR at 95%CI	AOR at 95%CI
	Good	Poor		
**Age**				
18–29 years ^**R**^	140	359	1.00	1.00
30–44 years	79	172	1.185 (0.85, 1.65)	1.06 (0.71, 1.58)
≥ 45 years	13	82	0.407 (0.22, 0.75)	0.31 (0.15, 0.62)*
**Sex**				
Male ^**R**^	113	200	1.00	1.00
Female	119	413	0.51 (0.37, 0.69)	0.50 (0.35, 0.71)*
**Occupation**				
Private/ merchant ^**R**^	82	82	1.00	1.00
Government employed	57	89	1.81 (1.19, 2.75)	1.40 (0.89, 2.20)
Student	49	152	0.91 (0.61, 1.37)	0.88 (0.55, 1.41)
Unemployed	44	140	0.88 (0.58, 1.35)	1.01 (0.65, 1.58)
**Marital status**				
Married ^**R**^	108	260	1.00	1.00
Single	107	283	0.91 (0.66, 1.24)	0.77 (0.52, 1.14)
Widowed/divorced	17	70	0.58 (0.32, 1.03)	0.64 (0.34, 1.18)
**Level of education**				
No formal education ^**R**^	40	126	1.00	1.00
Primary	129	100	0.91 (0.52, 1.52)	0.89 (0.49, 1.61)
Secondary	69	207	1.05 (0.67, 1.64)	1.02 (0.62, 1.68)
Diploma and above	94	178	1.66 (1.07, 2.56)	1.39 (0.84, 2.30)
**Knowledge about cancer risks**				
Poor ^R^	165	502	1.00	1.00
Good	65	106	1.86 (1.30, 2.65)	1.66 (1.14, 2.42)*
**Family member with cancer**				
Yes	47	67	2.07 (1.37, 3.11)	1.68 (1.07, 2.62)*
No ^R^	185	546	1.00	1.00

COR: Crude Odds Ratio; AOR: Adjusted Odds Ratio; P-value; probability value;

* P-value less than 0.05; R-reference category.

## Discussion

In Ethiopia, awareness creation and behavioral change communication are the pivots for the early detection, prevention, and control strategies of cancer [16]. The current study determined the level of cancer preventive practices. This study revealed that 27.5% of the participants had good preventive practices. In this study, nearly half of the respondents were aware of the warning symptoms of cancer. Of which, unexplained lump/swelling, not healing sore, unexplained bleeding, and persistent unexplained pain were the most commonly mentioned warning symptoms.

Eating fatty and processed meat, being overweight, not eating fruits and vegetables, and physical inactivity were fewer recognized risk factors of cancer by the respondents. The current findings were supported by other studies [17–19].

In addition, this study revealed only 20.2% of the respondents knew about cancer prevention. Physical exercise, adequate sleep time, and self-breast examination were the commonly mentioned cancer preventive practices by the respondents. Thus, huge tasks are needed to improve the cancer awareness and preventive practices of the community through health education and behavioral change communication.

The current study also identified the factors affecting cancer-preventive practice in Bahir Dar city. Older age, female sex, family member with cancer, and knowledge about cancer risks were determinants of cancer preventive practices.

For older ages (> 45 years), the odds of cancer preventive practice were 69% lower compared with younger ages. This finding was against the study done in India [20]. This discrepancy could be differences in the study population, period, and socio-cultural characteristics. The other possible explanation could be the current study was done in both sexes and on all cancer preventive practices. However, the Indian study was done on cervical-cancer related issues among the female population.

Likewise, for those female respondents, the odds of cancer preventive practice were 50% lower compared with male respondents. This finding was in line with the study done in high, middle, and low-income countries [2]. This finding could be justified majority of the respondents were females, and nearly 20% of them were not attended formal education. This could lead the respondents to less possibility of utilizing various social media, including internet access. In the meantime, females are busy with home-based tasks and might not give attention to cancer preventive practices. Hence, programs should target both females and males. Because if males are aware of females’ challenges, may encourage females to seek cancer prevention services.

Family member with cancer was the other determinant factor significantly associated with cancer preventive practices. For those participants whose family members had cancer, the odds of cancer preventive practices were 1.7 times higher compared with those who had not. This finding was in agreement with the study done in Iran and two studies in Saudi Arabia [5,10,11]. The possible justification for this could be some forms of cancer are a family or hereditary transfer [21], and the other family members would be worried about the complications of cancer suffering family member. And they have the likelihood of accessing information about the risk factors and preventive modalities of cancer, which would help them to implement cancer-preventive actions.

Moreover, this study also identified those participants who had good knowledge about cancer risk factors were 1.7 times higher compared with those who had poor knowledge. This finding is in line with the study findings of Iran, Ethiopia, Saudi, South Africa, and India, respectively [5,8,10,11,19,20]. Having better knowledge and understanding of cancer could help to implement cancer-preventive practices at the household level.

This study implicated that public health practitioners, policymakers, and other concerned stakeholders should have to strengthen cancer-related health education and behavioral change communications in the community to enhance the awareness of cancer risk factors and to support them implement the cancer-preventive practice.

### Strength of the study

Being a larger sample-sized, community-based, and both sexes included study make the result inferable to the community of Bahir Dar city.

### Limitations of the study

The cross-sectional nature (chicken-egg dilemma) of the study and recall and information bias was the weakness of this study. The study didn’t identify the other possible barriers (like age restrictions for screening/examination services, cost of fruits and vegetables) to uptake cancer preventive practices.

## Conclusion

Knowledge about cancer risks and good preventive practices was low. Family member with cancer and knowledge about cancer risks had higher odds of cancer preventive practices. Conversely, older age and female sex had lower odds of cancer preventive practices. This finding underscores the important interventions to enhance cancer awareness and preventive practices.

## Supporting information

S1 File (DOCX)

S2 File (SAV)

## Abbreviations

CAM: Cancer Awareness Measure
CLD: Chronic Liver Disease
CT: Computed Tomography
HBV: Hepatitis B Virus
HCV: Hepatitis C Virus
HPV: Human Papilloma Virus
IARC: International Agency for Research on Cancer
MRI: Magnetic Resonance Imaging
NCDs: Non Communicable Diseases
PET: Positron Emission Tomography
SEP: Socio-Economic Position
SPET: Single Photon Emission Tomography
UK: United Kingdom
WCRF: World Cancer Research Fund and WHO: World Health Organization

## References

[1] Bray F., et al., Global cancer statistics 2018: GLOBOCAN estimates of incidence and mortality worldwide for 36 cancers in 185 countries. CA: a cancer journal for clinicians, 2018. 68(6): p. 394–424. 30207593 10.3322/caac.21492

[2] De Souza J.A., et al., Global health equity: cancer care outcome disparities in high-, middle-, and low-income countries. Journal of Clinical Oncology, 2016. 34(1): p. 6. doi: 10.1200/JCO.2015.62.2860 26578608 PMC5795715

[3] Grosso G., et al., Possible role of diet in cancer: systematic review and multiple meta-analyses of dietary patterns, lifestyle factors, and cancer risk. Nutrition reviews, 2017. 75(6): p. 405–419. doi: 10.1093/nutrit/nux012 28969358

[4] Woldeamanuel Y.W., Girma B., and Teklu A.M., Cancer in Ethiopia. The Lancet. Oncology, 2013. 14(4): p. 289–290. doi: 10.1016/S1470-2045(12)70399-6 23561741

[5] Feizi A., et al., Public awareness of risk factors for cancer and its determinants in an Iranian population. Asia Pacific Journal of Public Health, 2010. 22(1): p. 76–88. doi: 10.1177/1010539509350768 20032037

[6] Nagai H. and Kim Y.H., Cancer prevention from the perspective of global cancer burden patterns. Journal of thoracic disease, 2017. 9(3): p. 448. doi: 10.21037/jtd.2017.02.75 28449441 PMC5394024

[7] Al-Azri M., et al., Awareness of risk factors for cancer among Omani adults-a community based study. Asian Pac J Cancer Prev, 2014. 15(13): p. 5401–5406. doi: 10.7314/apjcp.2014.15.13.5401 25041009

[8] Aweke Y.H., Ayanto S.Y., and Ersado T.L., Knowledge, attitude and practice for cervical cancer prevention and control among women of childbearing age in Hossana Town, Hadiya zone, Southern Ethiopia: Community-based cross-sectional study. PLoS One, 2017. 12(7). doi: 10.1371/journal.pone.0181415 28742851 PMC5526548

[9] Ebu N.I., et al., Knowledge, practice, and barriers toward cervical cancer screening in Elmina, Southern Ghana. International journal of women’s health, 2015. 7: p. 31. doi: 10.2147/IJWH.S71797 25565902 PMC4284003

[10] Ravichandran K., Al-Hamdan N.A., and Mohamed G., Knowledge, attitude, and behavior among Saudis toward cancer preventive practice. Journal of Family and Community Medicine, 2011. 18(3): p. 135. doi: 10.4103/2230-8229.90013 22175041 PMC3237202

[11] Ravichandran K., Mohamed G., and Al-Hamdan N.A., Public knowledge on cancer and its determinants among Saudis in the Riyadh Region of Saudi Arabia. Asian Pac J Cancer Prev, 2010. 11(5): p. 1175–1180. 21198259

[12] Timotewos G., et al., First data from a population based cancer registry in Ethiopia. Cancer epidemiology, 2018. 53: p. 93–98. doi: 10.1016/j.canep.2018.01.008 29414637

[13] Begoihn M., et al., Cervical cancer in Ethiopia–predictors of advanced stage and prolonged time to diagnosis. Infectious agents and cancer, 2019. 14(1): p. 36. doi: 10.1186/s13027-019-0255-4 31737087 PMC6849163

[14] FMoH E., National Cancer Control Plan 2016–2020. Addis Ababa DISEASE PREVENTION AND CONTROL DIRECTORATE, editor. DIRECTORATE DPAC, 2015.

[15] administration, B.D.c., Population and household survey. 2017/18. 2019.

[16] Munishi O.M., et al., Awareness of cancer risk factors and its signs and symptoms in Northern Tanzania: a Cross-Sectional Survey in the General Population and in People Living with HIV. Journal of Cancer Education, 2020. 35(4): p. 696–704. doi: 10.1007/s13187-019-01513-6 30915669 PMC7363667

[17] Opoku S.Y., Benwell M., and Yarney J., Knowledge, attitudes, beliefs, behaviour and breast cancer screening practices in Ghana, West Africa. Pan African Medical Journal, 2012. 11(1). PMC3325066 22514762

[18] Ramaiah R. and Jayarama S., Knowledge, attitude and practices about cervical cancer among rural married women: a cross sectional study. Int J Community Med Public Health, 2018. 5(4): p. 1466–1470.

[19] Ramathuba D.U., Ratshirumbi C.T., and Mashamba T.M., Knowledge, attitudes and practices toward breast cancer screening in a rural South African community. Curationis, 2015. 38(1): p. 1–8. 10.4102/curationis.v38i1.1172 PMC6091642 26017152

[20] Bora K., et al., Assessing the awareness level of breast and cervical cancer: a cross-sectional study in northeast India. International Journal of Medical Science and Public Health, 2016. 5(10): p. 1.

[21] Liu L., et al., Correlation between family history and characteristics of breast cancer. Scientific Reports, 2021. 11(1): p. 1–12. 33737705 10.1038/s41598-021-85899-8 PMC7973811

