# Supplementary material for: Cancers preventive practice and the determinants in Amhara regional state, Northwest Ethiopia
Source: PLoS One. 2022 May 19;17(5):e0267189. doi: 10.1371/journal.pone.0267189 (PMC9119519; doi:10.1371/journal.pone.0267189)
Supplement: S1 File — (DOCX) [file pone.0267189.s001.docx]

# English Version Questionnaire

**Information sheet for assessment of awareness and preventive practice toward cancer among Bahir Dar city residents**

You are being invited to take part in a research study. Before you decide whether or not to take part, it is important for you to understand why the research is being done and what it will involve. Please take time to read the following information carefully.

**What is the purpose of the study?**

Nursing graduate students of **GAMBY MEDICAL AND BUSINESS COLLEGE** is carrying out a survey to assess awareness of risk factors, signs and symptoms and preventive practice toward cancer. The results will be used to support the objectives of Ethiopian cancer control plan 2016-2020.

**Why have I been invited to take part?**

You have been chosen at random in which we are asking everyone aged between 18- 64.

**Do I have to take part?**

It is up to you to decide whether or not to take part, taking part is voluntary. If you do decide to take part you will be given this information sheet to keep and be asked to sign a consent form. If you decide to take part you are still free to withdraw at any time and without giving a reason.

**What would I have to do?**

If you decide to take part, the survey will take approximately 20 minutes to complete.

**Confidentiality**

All the information that is collected will be anonymous and kept strictly confidential. Your personal data will be held in accordance with the Data Protection Act 1998.

**What happens to the information that is collected?**

All details that can identify you will be removed before storing the data. All the information collected in this survey (although not your name), will be stored in the UK Data Archive, which is a secure national bank where the results of many surveys are kept.

**Consent form for assessment of preventive practice toward cancer among Bahir Dar city residents**

*Please tick the appropriate boxes*

- I have read and understood the project information sheet dated DD/MM/YYYY.
- I have been given the opportunity to ask questions about the project.
- I agree to take part in the project. Taking part in the project will include completing a survey.
- I understand that my taking part is voluntary; I can withdraw from the study at any time and I will not be asked any questions about why I no longer want to take part.
- I understand my personal details such as phone number and address will not be revealed to people outside the project.
- I understand that my words may be quoted in publications, reports, web pages, and other research outputs but my name will not be used unless I requested it above.
- I understand that other researchers will have access to this data only if they agree to preserve the confidentiality of that data and if they agree to the terms I have specified in this form.
- I understand that other researchers may use my words in publications, reports, web pages, and other research outputs according to the terms I have specified in this form.

_____________________________ ______________ ____________________

Name of participant Signature Date

_____________________________ ______________ ____________________

Name of participant Signature Date

| **Part 1 socio-demographic questions** | | |
| --- | --- | --- |
| **Question** |  | **Jump** |
| Q101. What is your age? | _______________ |  |
| Q102. What is your gender? | 1. Male 2. Female |  |
| Q103. What is your ethnic group? | 1. Amhara 2. Oromo 3. Tigre 4. Other |  |
| Q104. What is your religion? | 1. Orthodox Tewahido 2. Non-Orthodox |  |
| Q105. What is your marital status? | 1. Married 2. Unmarried 3. Divorced 4. Widowed |  |
| Q106. What is your educational status? | 1. Unable to read and write 2. Able to read and write only 3. Primary school 4. Secondary school 5. College and above |  |
| Q107. Your living arrangement | 1. Private House 2. Government house 3. Rental house 4. Living with family/friends |  |
| Q108. What is your occupational status? | 1. Private employee 2. Government employee 3. Merchant 4. Student 5. House wife 6. Unemployed 7. Retired |  |
| Q109. Have You or other family members had cancer? | 1. yes 2. No | 2>>110 |
| Q110. If yes who had it? | 1. Me 2. Partner 3. Close family member 4. Other family member 5. Close friend |  |
| Q111. When you get diseased, where is your usual place of choice? | 1. Private health facility 2. Government health facility 3. Holy water 4. I do not go anywhere 5. Explain if any other____________ |  |

| **Part 2 questionnaire about cancer awareness** | | |
| --- | --- | --- |
| **Questions** |  | **jump** |
| Q201. Is cancer communicable disease? | 1. Yes 2. No | **2>>203** |
| Q202. If yes, through which route does it transmitted? | 1. Blood contact 2. Sexual contact 3. Eating together with diseased person 4. Air droplets 5. Contaminated food or drink |  |

Q203. The following may or may not be warning signs for cancer. We are interested in your opinion:

|  | Yes | No | Don’t know |
| --- | --- | --- | --- |
| Q2031. Do you think unexplained bleeding could be a sign of cancer? |  |  |  |
| Q2032. Do you think a persistent cough or hoarseness could be a sign of cancer? |  |  |  |
| Q2033. Do you think a persistent change in bowel or bladder habits could be a  Sign of cancer? |  |  |  |
| Q2034. Do you think persistent difficulty swallowing could be a sign of cancer? |  |  |  |
| Q2035. Do you think a change in the appearance of a mole could be a sign of Cancer? |  |  |  |
| Q2036. Do you think a sore that does not heal could be a sign of cancer? |  |  |  |
| Q2037. Do you think an unexplained lump or swelling could be a sign of cancer? |  |  |  |
| Q2038. Do you think persistent unexplained pain could be a sign of cancer? |  |  |  |
| Q2039. Do you think unexplained weight loss could be a sign of cancer? |  |  |  |

**Q204. These are some of the things that can increase a person’s chance of developing cancer. How much do you agree that each of these can increase a person’s chance of developing cancer?**

|  | Strongly disagree | Disagree | Not sure | Agree | Strongly agree |
| --- | --- | --- | --- | --- | --- |
| Q2041. Smoking any cigarettes at all |  |  |  |  |  |
| Q2042. Exposure to another person’s cigarette smoke |  |  |  |  |  |
| Q2043. Drinking more than 1 unit of alcohol a day |  |  |  |  |  |
| Q2044. Eating less than 5 portions of fruit and vegetables a day |  |  |  |  |  |
| Q2045. Eating red or processed meat once a day or more |  |  |  |  |  |
| Q2046. Being overweight (BMI over 25) |  |  |  |  |  |
| Q2047. Getting sunburnt more than once as a child |  |  |  |  |  |
| Q2048. Being over 70 years old |  |  |  |  |  |
| Q2049. Having a close relative with cancer |  |  |  |  |  |
| Q20410. Infection with HPV (Human Papillomavirus) |  |  |  |  |  |
| Q20411. Doing less than 30 min of moderate physical activity 5 times a week |  |  |  |  |  |
| Q20412. Chronic infection with hepatitis B or C virus |  |  |  |  |  |

| **Questions About preventive practice of cancer** | | |
| --- | --- | --- |
| Questions |  | jump |
| Q301. What Is Your Current Weight? | ___________________ |  |
| Q302. How Much Exercise Do You Get (Work Or Play)? | 1. None 2. Slight 3. Moderate 4. Heavy |  |
| Q303. How Many Hours Of Sleep Do You Usually Get a Night? | 1. Less Than 6 Hours 2. 6-8 Hours 3. Greater Than 8 Hours |  |
| Q304. Have You Ever Smoked Cigarette Regularly? | 1. Yes 2. No | 2>>307 |
| Q305. Do You Currently Smoke Cigarette | 1. Yes 2. No | 2>>307 |
| Q306. If You Currently Smoke Cigarette, How Many Cigarette Do You Usually Smoke A Day? | ___________________ |  |
| Q307. On Average, How Often Did You Drink Beer, Wine Or Liquor In The Last Year? | 1. Never or < 1 Day/Month 2. 1-4 Days/Month 3. 2-5 Days/Week 4. 6-7 Days/Week |  |
| Q308. On Average, On The Days That You Drank Beer, Wine, Or Liquor, How Many Drinks Did You Have? | 1. I Don’t Drink Alcohol 2. 1 Drink/Day 3. 2 Drinks/Day 4. 3 Or More Drinks/Day |  |
| Q309. When Eating Meat, Do You Avoid Eating The Fat? | 1. yes 2. No |  |
| Q310. Do You Often Add Salt To Your Food? | 1. yes 2. No |  |
| Q311. In Your current or previous Work Have You Ever Been Exposed To Gases, Dusts, Fumes, Chemicals, and Oils Or Solvents Of Any Sort Which Might Possess Effect To Your Lungs? | 1. yes 2. No |  |
| Q312. If Yes, For Which of Them? You Can Choose More Than One Exposure If You Have Been. | 1. Chemicals 2. Textile Dusts 3. Wood Dusts 4. Gases 5. Solvents 6. Oils |  |
| Q313. Have You Ever Worked With X-Rays Or Radioactive Materials? | 1. yes 2. No |  |

*If You Are Female Please Skip To Question 21. If You Are Male Please Answer The Following.* Q314. In The Past Two Years, Have You Had Any of The Following? (If Yes, Mark All That Apply.)

|  | Yes, For Routine Exams | Yes, For Symptoms | No |
| --- | --- | --- | --- |
| Q3141. Colonoscopy |  |  |  |
| Q3142. Sigmoidoscopy |  |  |  |
| Q3143. A Physical Exam |  |  |  |
| Q3144. Blood Analysis |  |  |  |

*If You Are Female Please Answer The Following*

Q315. Do You Regularly Perform Breast Self-Examination?

1. Yes
2. No

Q316. Have You Vaccinated Against Human Papilloma Virus?

1. Yes
2. No

Q317. In The Past Two Years, Have You Had Any of The Following? (If Yes, Mark All That Apply.)

|  | Yes, For Routine Exams | Yes, For Symptoms | No |
| --- | --- | --- | --- |
| Q3171. Colonoscopy |  |  |  |
| Q3172. Sigmoidoscopy |  |  |  |
| Q3173. A Physical Exam |  |  |  |
| Q3174. Blood Analysis |  |  |  |
| Q3175. Pap Smear |  |  |  |
| Q3176. Mammogram |  |  |  |

**Description**

- ***Colonoscopy****: A Long Tube Inserted Into the Rectum to Examine the Entire Colon for Cancer or Other Problems. A Medicine was given through a Needle in Your arm to Make You Sleepy and Someone Else Needed to Drive You Home.*
- ***Sigmoidoscopy****: A Short Tube Inserted Into Rectum to Examine the Lower Part of the Colon to Check for Cancer or Other Problems. You Were Awake And Not Given A Medicine To Make You Sleepy. You Were Probably Able To Drive Yourself Home*
- ***Slight Physical Activity****: includes very slow and normal walks*
- ***Moderate Physical Activity****: includes fast paced walk, bike ride, or any activity where sweating by the end.*
- ***Heavy Physical Activity****: includes running, increased heart rate, sweating and heavy breathing.*

# የአማርኛጥያቄዎች

**የህብረተሰቡንየካንሰርግንዛቤእናየመከላከልተግባርንበባህርዳርከተማነዋሪዎችላይየሚያጠናውጥናትየመረጃገጽ**

አርስዎእዚህጥናትላይእንዲሳተፉተጋብዘዋል፡፡ለመሳተፍከመወሰኖበፊትግንጥናቱለምንእንደተደረገእናምንላይእንደሚያውጠነጥንመረዳትጠቃሚነው፡፡ስለዚህእባክዎየሚመለከተውንመረጃአስተውለውበጥንቃቄያንብቡት፡፡

**የጥናቱአላማምንድንነው?**

የጋምቢህክምናእናቢዝነስኮሌጅተመራቂየነርሲንግተማሪዎትየማህበረሰቡንግንዛቤስለካንሰርአጋላጭሀኔታዎች፣ምልክቶችእናየመከላከልተግባርለማወቅጥናትእያካሄዱነው፡፡የጥናቱውጤትየኢትዮጵያንየካንሰርመቆጣጠርእቅድ 2016-2020 የሚደግፍይሆናል፡፡

**እኔእንድሳተፍለምንተጋበዝኩ?**

እርስዎየጥናቱአካልሆነውየተመረጡትየጥናቱንመስፈርትያሚያሙሉስለሆነእናበእድልነው፡፡

**መሳተፍአለብኝ?**

መሳተፍወይምአለመሳተፍየእርስዎውሳኔነው፡፡ተሳትፎውበፈቃደኝነትላይየተመሰረተነው፡፡ለመሳተፍከወሰኑየመረጃእናየመስማሚያቅጽእንዲፈርሙይሰጥዎታል፡፡በተጨማሪምለመሳተፍከወሰኑበማንኛውምስዓትምንምምክንያትመስጠትሳይጠበቅብዎጥናቱንማቋረጥይቻላል፡፡

**ምንማድረግአለብኝ?**

ለመሳፈትከወሰንክ/ሽጥናቱንለመጨረስከ 15-20 ደቂቃይወስዳል፡፡

**ሚስጠራዉነቱ**

ሁሉምየተሰበሰበመረጃማንምየማያውቀውእናሚስጥራዉነቱምበጥብቅየተጠበቀይሆናል፡፡የግልመረጃህ/ሽየተጠበቀይሆናል፡፡

**የተሰበሰበውመረጃምንይሆናል?**

የአንተን/ችንማንነትሊገልጽውየሚችሉሁሉምመረጃዎችከመጠራቀሙበፊትይወገዳሉ፡፡በጥያቄውየተሰጠኸው /ሽውመልስብቻአጠቃላይውጤትለማግኘትእንጠቀምበታለን፡፡

**የካንሰርግንዛቤእናየመከላከልትግበራጥናትየመስማሚያቅጽ**

- የጥናቱንየመረጃወረቀትአንብቤተረድቻለሁ፡፡
- ስለጥናቱጥያቄልጠይቅእድሉተሰጥቶኝነበር፡፡
- ጥናቱላይለመሳተፍተስማምቻለሁ፡፡ጥናቱላይመለመሳተፍመስማማትእነዚህንጥያቄዎችመሙላትንያካትታል፡
- ተሳትፎየበእኔፈቃደኝነትላይየተመሠረተእንደሆነተረድቻለሁ፡፡በማንኛውምሰዓትጥናቱንአቋርጨመውጣትየምችልሲሆንለምንአቋርጨእንደወጣሁአልጠየቅም፡፡
- ሌሎችጥናትአጥኝዎችይህንመረጃማግኘትየሚችሉትየጥናቱንሚስጥራዊነትለመጠበቅከተስማሙብቻነው፡፡ከዚህወረቀትላይእኔከጠቀስኩትቃላቶችጋርመስማማትከቻሉብቻእንደሆነተረድቻለሁ፡፡
- ሌሎችጥናትአጥኝዎችየእኔንቃላቶችለተለያዩዘገባዎች፣ድህረገጾችእንዲሁምሌሎችየጥናትውጤቶችላይሊጠቀሟቸውእንደሚችሉተረድቻለሁ፡፡

_____________________________ ______________ ____________________

የአጥኚውስምፊርማቀን

_____________________________ ______________ ____________________

የተሳታፊውስምፊርማቀን

| **ክፍል 1 የማህበራዊጥያቄዎች** | | |
| --- | --- | --- |
| **ጥያቄ** |  | **እለፍ** |
| Q101. እድሜህ/ሽስንትነው? (በአመት) | ______________ |  |
| Q102. ፆታህ/ሽምንድንነው ? | 1. ወንድ 2. ሴት |  |
| Q103. ብሔርህ/ሽምንድንነው ? | 1. አማራ 2. ኦሮሞ 3. ትግሬ 4. ሌላ |  |
| Q104. ሀይማኖትህ/ሽምንድንነው ? | 1. ኦርቶዶክስተዋህዶ 2. ሙስሊም 3. ፕሮቴስታንት 4. ካቶሊክ 5. ሌላ |  |
| Q105. የጋብቻህ/ሽሁኔታምንድንነው ? | 1. ያገባ 2. ያላገባ 3. አግብቶየፈታ/ች 4. የሞተበት/ባት |  |
| Q106. ያለህ/ሽየትምህርትደረጃ | 1. ማንበብእናመጻፍየማይችል 2. ማንበብእናመጻፍብቻየሚችል 3. የመጀመሪያደረጃድረስየተማረ 4. ሁለተኛደረጃድረስየተማረ 5. ኮሌጅእናከዛበላይየተማረ |  |
| Q107. የቤትሁኔታህ/ሽምንድንነው? | 1. የግል 2. የመንግስት 3. የኪራይ 4. ከቤተሰብወይምከጋደኛጋርየሚኖር |  |
| Q108. የስራሁኔታህ/ሽምንድንነው ? | 1. የግልሰራተኛ 2. የመንግስትሰራተኛ 3. ነጋዴ 4. ተማሪ 5. የቤትእመቤት 6. ስራየሌለው 7. ጡረታየወጣ |  |
| Q109. አንተ/አንችወይምከቤተሰብአባላቶችህበካንሰርየተያዘሰውአለ ? | 1. አለ 2. የለም | **2››Q111** |
| Q110. መልሱአዎከሆነማንነውየተያዘው? | 1. እኔ 2. ባለቤቴ 3. የቅርብቤተሰብ 4. ሌላየቤተሰብአባል 5. የቅርብጓደኛ |  |
| Q111. ስትታመም/ሚህክምናለማግኘትአብዛኛውንጊዜየምትሄደው/የምትሄጅውየትነው? | 1. የግልጤናድርጅት 2. የመንግስትጤናድርጅት 3. ጸበል 4. የትምአልሄድም 5. ሌላካለይገለጽ_______________ |  |

| **ክፍል 2 የካንሰርግንዛቤጥያቄዎች** | | |
| --- | --- | --- |
| **ጥያቄ** |  | **እለፍ** |
| Q201. ካንሰርተላላፊበሽታነው? | 1. ነው 2. አይደለም | **2››Q203** |
| Q202. አዎካሉበምንይተላለፋል? | 1. በደምንክኪ 2. በግብረስጋግንኙነት 3. አብሮበመመገብ 4. በትንፋሽ 5. በተበከለምግብ /መጠጥ |  |

**Q210.የሚከተሉትየካንሰርቅድመምልክቶችሊሆኑምላይሆኑምይችላሉ፤የአንተን/የአንቺንየግልአስተያየትስጥ/ስጭ::**

|  | **አዎ** | **አይደለም** | **አላውቅም** |
| --- | --- | --- | --- |
| Q2101. ምክንያቱያልታወቀየደምመፍሰሰየካንሰርቅድመምልክትይመስለዎታል ? |  |  |  |
| Q2102. ምክንያቱያልታወቀቀጣይነትያለውሳልእናየድምጽመጐርነንየካንሰርቅድመምልክትነውብለውያስባሉ ? |  |  |  |
| Q2103. የተዛባቀጣይነትያለውየሰገራእናየሽንትቤትልምድሂደትለውጥየካንሰርቅድመምልክትይመስልዎታል ? |  |  |  |
| Q2104. ቀጣይነትያለውለመዋጥመቸገርየካንሰርቅድመምልክትይመስለወታል? |  |  |  |
| Q2105. በሰውነትላይየሚታዩጥቋቁርነጠብጣቦች (በተለምዶማርያምየሣመችኝየሚባለው) ቅርፅመቀየርየካንሰርቅድመምልክትሊሆንይችላል ? |  |  |  |
| Q2106. ለረጅምጊዜየማይድንቁስልየካንሰርቅድመምልክትሊሆንይችላል ? |  |  |  |
| Q2107. ምክንያቱያልታወቀእብጠትየካንሰርቅድመምልክትሊሆንይችላል ? |  |  |  |
| Q2108. ምክንያቱያልታወቀእናቀጣይነትያለውህመምየካንሰርቅድመምልክትሊሆንይችላል? |  |  |  |
| Q2109. ምክንያቱያልታወቀየክብደትመቀነስየካንሰርቅድመምልክትሊሆንይችላል ? |  |  |  |

**Q211.ከዚህበታችየተዘረዘሩትአጋላጭሁኔታዎችአንድንሰውበካንሰርየመያዝእድልይጨምራሉ፡፡በነዚህነገሮችእርስዎምንያህልይስማማሉ ?**

|  | **በጣምአልስማማም** | **አልስማማም** | **እርግጠኛአይደለሁም** | **እስማማለሁ** | **በጣምእስማማለሁ** |
| --- | --- | --- | --- | --- | --- |
| Q2111. ሲጋራማጨስ |  |  |  |  |  |
| Q2112. ሌሎችሰዎችለሚያጨሱትሲጋራመጋለጥ |  |  |  |  |  |
| Q2113. በቀንውስጥከአንድጠርሙስበላይየአልኮልመጠጥመጠቀም |  |  |  |  |  |
| Q2114. በቀንከ5 መጠንያነሰአረንጓዴአትክልትናፍራፍሬመመገብ |  |  |  |  |  |
| Q2115. በቀንውስጥአንድጊዜወይምከዛበላይጥሬወይምበፋብሪካየተመረተስጋመመገብ |  |  |  |  |  |
| Q2116. ከመጠንበላይየሆነክብደት*(የሰውነትክብደትምጣኔማሳያ)* /BMI > or = 25/ |  |  |  |  |  |
| Q2117. በልጅነትጊዜከአንድጊዜበላይለፀሐይቃጠሎመጋለጥ |  |  |  |  |  |
| Q2118. እድሜከ7ዐዓመትበላይመሆን |  |  |  |  |  |
| Q2119. በካንሰርበተያዘየቅርብዘመድመኖር |  |  |  |  |  |
| Q21110. የማህጸንበርጫፍንበሚያጠቃቫይረስ*(ሂውማንፓፒሎማቫይረስ)*መወረርወይምመያዝ |  |  |  |  |  |
| Q21111. በሣምንትውስጥከ5 ጊዜበታችለ3ዐደቂቃመካከለኛየአካልብቃትእንቅስቃሴመስራት |  |  |  |  |  |
| Q21112. ስርበሰደደየጉበትቫይረስቢወይምሲመወረርወይምመያዝ |  |  |  |  |  |

| **ክፍል 3 ካንሰርንየመከላከልልምድንበተመለከተጥያቄዎች** | | |
| --- | --- | --- |
| **ጥያቄ** |  | **እለፍ** |
| Q301. አሁንላይየሰውነትክብደትህ/ሽስንትነው ? | ________________________ |  |
| Q302. ምንያህልየሰውነትእንቅስቃሴትሰራለህ/ትሰሪያለሽ? | 1. ምንም 2. ትንሽ 3. መካከለኛ 4. ከባድ |  |
| Q303. አብዛኛዉንጊዜማታላይለምንያህልሰአትትተኛለህ/ሽ? | 1. ከ 6 ሰአትያነሰ 2. ከ 6-8 ሰአት 3. ከ 8 ሰአትበላይ |  |
| Q304. ሲጋራአጭሰህ/ሽታውቃለህ/ሽ? | 1. አውቃለሁ 2. አላውቅም | **2››Q307** |
| Q305. አሁንላይሲጋራታጨሳለህ/ሽ? | 1. አጨሳለሁ 2. አላጨስም | **2››Q307** |
| Q306. አሁንላይሲጋራየምታጨስ/ሽከሆነበቀንምንያህልሲጋራታጨሳለህ/ሽ? | ___________________ |  |
| Q307. ባለፈውአመትበአማካኝለምንያህልጊዜቢራ፤ወይንወይምሌላየአልኮልመጠጥጠጥተሃል/ጠጥተሻል ? | 1. ምንምወይምበወርከ 1 ጊዜበታች 2. በወርከ 1- 4 ጊዜ 3. በሳምንትከ 3-5 ቀናት 4. በሳምንትከ 6-7 ቀናት |  |
| Q308. ቢራ፤ወይንወይምሌላየአልኮልመጠጥየጠጣህጊዜበአማካኝምንያህልነበርየጠጣህው? | 1. አልኮልአልጠጣም 2. በቀን 1 መጠጥ 3. በቀን 2 መጠጥ 4. በቀን 3 መጠጥወይምከዛበላይ |  |
| Q309. ስጋስትበላ/ስትበይስቡንታስወግደዋለህ/ለሽ? | 1. አስወግዳለሁ 2. አላስወግድም |  |
| Q310. አብዛኛውንጊዜምግብህ/ሽላይጨውትጠቀማለህ/ሽ? | 1. እጠቀማለሁ 2. አልጠቀምም |  |
| Q311. በአሁንወይምበበፊትስራህ/ሽለጋዝ፣ለአቧራ፣ለኬሚካሎች፣ለኬሚካልመበጥበጫዎችወይምሌሎችተጋልጠህ/ሽታውቂያለሽ? | 1. አውቃለሁ 2. አላውቅም | **2››313** |
| Q312. አውቃለሁካልሽ/ካልክለየትኞቹ ? ከአንድበላይመምረጥይቻላል:: | 1. ኬሚካሎች 2. የጨርቃጨርቅብናኖች 3. የእንጨትብናኖች 4. ጋዞች 5. የኬሚካልማቅጠኛዎች 6. ቅባቶች |  |
| Q313. ከራጅወይምከጨረረአመንጪነገሮችጋርሰርተህ/ሽታውቃለህ/ሽ ? | 1. አውቃለሁ 2. አላውቅም |  |

***እባክዎሴትከሆኑወደጥያቄቁጥር 315 ይለፉ:: ወንድከሆኑየሚከተሉትንጥያቄዎችይመልሱ***

**Q314. ባለፉትሁለትአመታትውስጥከታችከተዘረዘሩትምርመራዎችውስጥየትኛውንአድርገሀል?**

|  | **አዎለተለመደምርመራ** | **አዎለበሽታምልክቶች** | **አላውቅም** |
| --- | --- | --- | --- |
| Q3141. በፊንጢጣበኩልበሚገባረጅምመሳሪያአንጀትንመታየት |  |  |  |
| Q3142. በፊንጢጣበኩልበሚገባአጭርመሳሪያአንጀትንመታየት |  |  |  |
| Q3143. አጠቃላይየሰውነትአካላትምርመራ |  |  |  |
| Q3144. ሙሉየደምምርመራ |  |  |  |

Q315. ጡትሽንበራስሽትትመረምሪያለሽ ?

1. እመረምራለሁ
2. አልመረምርም

Q316. የማህፀንጫፍካንሰርክትባትተከትበሻል ?

1. ተከትቤአለሁ
2. አልተከተብኩም

**Q317. ባለፉትሁለትአመታትውስጥከታችከተዘረዘሩትምርመራዎችውስጥየትኛውንአድርገሻል?**

|  | **አዎለተለመደምርመራ** | **አዎለበሽታምልክቶች** | **አላውቅም** |
| --- | --- | --- | --- |
| Q3171. በፊንጢጣበኩልበሚገባረጅምመሳሪያአንጀትንመታየት |  |  |  |
| Q3172. በፊንጢጣበኩልበሚገባአጭርመሳሪያአንጀትንመታየት |  |  |  |
| Q3173. አጠቃላይየሰውነትአካላትምርመራ |  |  |  |
| Q3174. ሙሉየደምምርመራ |  |  |  |
| Q3175. የማህፀንጫፍካንሰርቅድመምርመራ |  |  |  |
| Q3176. የጡትካንሰርቅድመምርመራ |  |  |  |

***መግለጫ***

- ***አንድመጠንአረንጓዴአትክልትወይምፍራፍሬ****የሚባለውአንድብርቱካንወይምሙዝወይምሌላአረንጋዴአትክልትእናፍራፍሬነው፡፡*
- ***ትንሽእንቅስቃሴየሚባለው****በጣምዝግ-ያለእናየተለመደአይነትመራመድንያካትታል፡፡*
- ***መካከለኛእንቅስቃሴየሚባለው****ፈጣንእርምጃን፣ብስክሌትመንዳትንወይምማንኛውንምላብሊያስወጣየሚችልእንቅስቃሴያካትታል፡፡*
- ***ከባድእንቅስቃሴየሚባለው****መሮጥን፣የልብምትመጨመርን፣ላበትንእናከባድአተነፋፈስየሚያመጡእቅስቃሴዎችንያካትታል፡፡*

***ስለትብብርዎበጣምእናመሰግናለን****!!!*
